# The study on the relationship between disease control status of children with asthma and caregiver psychological resilience and family function

**DOI:** 10.3389/fped.2026.1773771

**Published:** 2026-03-18

**Authors:** Limin Chen

**Affiliations:** Neonatal Department, Affiliated Hospital of Jiangnan University, Wuxi, Jiangsu, China

**Keywords:** asthma, caregivers, child, family relations, psychological resilience

## Abstract

**Background:**

Asthma is a common chronic disease in children, and its control level is influenced by multiple factors. The psychological state of caregivers and family function may play important roles. This study aimed to investigate the relationship between asthma control in children and caregiver psychological resilience and family function.

**Methods:**

This retrospective study included 112 children with asthma and their primary caregivers who were admitted to our hospital between January 2022 and December 2024. The children were divided into a controlled group (*n* = 60) and an uncontrolled group (*n* = 52) based on their childhood asthma control test (C-ACT) scores. Data collection involved general information questionnaires, the general health questionnaire (GHQ), the coping health inventory for parents (CHIP), the family assessment device (FAD), and the family management measure (FaMM). These assessments were conducted during the patients’ hospital visits.

**Results:**

Caregivers in the uncontrolled group had significantly lower self-affirmation scores (5.92 ± 0.84 vs. 6.35 ± 0.72, *P* = 0.004) but higher depression (2.05 ± 0.63 vs. 1.72 ± 0.45, *P* = 0.003) and anxiety scores (1.64 ± 0.51 vs. 1.38 ± 0.36, *P* = 0.003). They also had lower scores on CHIP subscales (Family, Support, Medical, all *P* < 0.05). Regarding family function, the uncontrolled group had significantly higher scores across all FAD dimensions (e.g., Problem-solving, Communication, all *P* < 0.05) and worse scores on most FaMM subscales (e.g., lower Child Acceptance, higher Disease Impact, all *P* < 0.05). Correlation analysis confirmed that poorer caregiver psychological resilience and worse family function were significantly associated with uncontrolled asthma.

**Conclusion:**

Poorer psychological resilience in caregivers and dysfunctional family dynamics are significantly associated with inadequate asthma control in children.

## 1 Introduction

Asthma is one of the most prevalent chronic respiratory diseases affecting children worldwide. Its management presents a significant challenge to global public health systems (1). The primary goal of asthma treatment is to achieve and maintain optimal clinical control. This control is defined by the minimization of daytime and nighttime symptoms, the reduction of exacerbation risk, and the preservation of normal lung function and physical activity levels (2, 3). Effective control is crucial for ensuring a high quality of life for the affected child and their family (4).

Despite the availability of effective pharmacological treatments and established clinical guidelines, a substantial proportion of pediatric asthma patients fail to achieve adequate disease control (5). The level of asthma control is not determined by medical management alone. Instead, it is influenced by a complex interplay of factors that extend beyond the clinical sphere (6). A comprehensive understanding of these determinants is essential for developing more effective and holistic management strategies. This perspective aligns with the biopsychosocial model of health, which emphasizes the interaction between biological, psychological, and social factors in disease progression and outcomes (7).

Within this biopsychosocial framework, the family environment serves as the primary context for a child's daily life and illness management. The family's functional dynamics, including problem-solving abilities, communication patterns, and emotional responsiveness, are critically important (8). A well-functioning family unit can provide stable support, consistently adhere to medication regimens, and effectively manage environmental triggers. Conversely, family dysfunction can disrupt these essential processes, potentially leading to poor treatment adherence and inadequate symptom monitoring, thereby compromising asthma control (9).

The primary caregiver, often a parent, is the key agent in the day-to-day execution of the child's asthma management plan. The immense and sustained responsibility of caregiving can be a significant source of stress and psychological burden. The caregiver's psychological resilience, or their capacity to adapt and cope with these challenges, is therefore a vital resource (10). A resilient caregiver is likely to maintain consistent disease management practices and a positive outlook, which may directly influence the child's health outcomes. However, the specific role of caregiver resilience, in conjunction with overall family function, in relation to the level of asthma control in children remains an area requiring further empirical investigation (11).

In recent years, research has gradually revealed the roles of psychological resilience and family functioning in pediatric asthma control. For example, Chong et al. (9) found that parental psychological flexibility influences self-efficacy in asthma management, while Han et al. (12) showed that poor family communication is associated with poorer control. Similarly, other studies on chronic diseases, such as diabetes, support the impact of family functioning on treatment adherence (13). However, existing literature predominantly focuses on single dimensions or Western populations, lacking a comprehensive assessment of multidimensional psychosocial factors, particularly with insufficient exploration of mechanisms within the Chinese context.

Therefore, to address these gaps, the primary objective of this study is to systematically investigate the relationship between the disease control status of children with asthma and the psychological resilience of their caregivers, as well as the overall functioning of their families. By employing a comprehensive set of instruments, this research aims to provide a holistic view of psychosocial determinants and identify modifiable factors for future interventions, thereby contributing to the biopsychosocial model of pediatric asthma management.

## 2 Materials and methods

### 2.1 Study subject

A retrospective analysis was conducted on 112 children with asthma and their parents who visited our hospital from January 2022 to December 2024. Inclusion criteria were: (1) meeting the diagnostic criteria outlined in the “Guidelines for Diagnosis and Optimal Treatment of Asthma in Children (2016)” (14); (2) having an asthma duration of ≥3 months; (3) being aged between 7 and 11 years old; (4) possessing normal intelligence and completing the responses to questions 1–4 of the childhood asthma control test (C-ACT); (5) having parents capable of normal communication and literacy; (6) complete medical records and survey questionnaires without any missing data. Exclusion criteria were: (1) children with other coexisting chronic diseases; (2) children in the acute exacerbation phase of asthma.

Based on the C-ACT scores, the 112 children with asthma were defined into a controlled group (*n* = 60) and an uncontrolled group (*n* = 52). The controlled group was defined as having a C-ACT score greater than 19, while the uncontrolled group was defined as having a C-ACT score of 19 or less.

### 2.2 Ethical statement

This study rigorously followed the requirements of the World Medical Association's Declaration of Helsinki (revised in 2022) and the Guidelines for Ethical Review of Medical Research. The research protocol has been reviewed and approved by the Ethics Committee of Affiliated Hospital of Jiangnan University. As this was a retrospective study, all data were sourced from the hospital's electronic medical record system and previously completed clinical questionnaires. No additional interventions were performed on the children or their caregivers during data collection, and all personal identifying information was anonymized. Therefore, the Ethics Committee exempted the requirement for informed consent.

### 2.3 Data collection

(1) General information: This included the children and their family situations. The children situation encompassed age, gender, body mass index (BMI), allergy history, disease duration, whether they attended regular follow-ups, whether they were an only child, and school attendance. The family situation included the identity of the primary caregiver, parents’ ages and education levels, family structure, monthly family income, and whether any family member smoking. BMI was calculated and converted to sex- and age-specific percentiles based on WHO Growth Standards. These general data were obtained retrospectively from structured fields in the electronic medical records or standardized forms completed during initial clinical assessments, ensuring no additional contact with participants.
(2) C-ACT: The questionnaire consisted of 7 items, with a maximum score of 27. Questions 1 to 4 were completed by the child, and questions 5 to 7 were completed by the parent, with the final score being the sum of all responses. A score of ≤19 indicated uncontrolled asthma, 20 to 22 indicated partially controlled asthma, and ≥23 indicated fully controlled asthma. The Cronbach's alpha coefficient for this scale is 0.759 (15).
(3) General health questionnaire (GHQ): The scale consisted of 20 items, divided into three subscales: self-affirmation (9 items), depression (6 items), and anxiety (5 items). The scale used a binary “yes/no” response format. Except for items 7 and 10, which were reverse-scored, all other items received a score of 1 for “yes” and 0 for “no”. Higher scores on the scale indicated higher levels of the respective dimension. The Cronbach's alpha coefficient for this scale is 0.88 (16).
(4) Coping health inventory for parents (CHIP): The scale consisted of 45 items, divided into three subscales: Family (family integration, cooperation, and optimistic definition of the situation) (15 items), Support (protective social support, self-esteem, and psychological stability) (15 items), and Medical (understanding medical conditions through communication with other parents and consulting the medical team) (15 items). Each item was rated on a 4-point Likert scale ranging from “not at all helpful” to “very helpful”, scored from 1 to 4. Higher scores indicated more frequent use of that particular coping strategy by the parents. The Cronbach's alpha coefficients for this scale range from 0.80 to 0.86 (17).
(5) Family assessment device (FAD): The scale consisted of 60 items, divided into seven subscales: problem-solving (6 items), communication (9 items), roles (10 items), affective responsiveness (10 items), affective involvement (10 items), behavioral control (9 items), and general functioning (12 items). Each item was rated on a 4-point scale ranging from “very much like my family” to “not at all like my family”, scored from 1 to 4. The final score for each subscale was the average of the total scores of its respective items. Higher scores on each dimension indicated poorer family functioning. The Cronbach's alpha coefficient for this scale is 0.86 (18).
(6) Family management measure (FaMM): The scale consisted of 53 items, divided into six subscales: child acceptance (7 items), disease impact (12 items), financial difficulties (5 items), disease burden (13 items), caregiving efficacy (8 items), and parental relationships (8 items). Each item was rated on a 5-point Likert scale ranging from “strongly agree” to “strongly disagree”, scored from 5 to 1. The final score for each subscale was the total score of its respective items. The Cronbach's alpha coefficient for this scale is 0.84 (19).

It is important to note that all questionnaire data (GHQ, CHIP, FAD, FaMM) were collected retrospectively from previously completed clinical assessments during the patients' hospital visits between 2022 and 2024, with no prospective data collection involved in this study.

### 2.4 Statistical analysis

Data were processed and analyzed using SPSS software (version 29.0; developed by SPSS Inc., Chicago, IL, USA). Based on the Shapiro–Wilk test, continuous variables in this study were assessed for normality. The test results indicated that all continuous variables (e.g., age, BMI percentile, questionnaire scores) did not significantly deviate from a normal distribution (Shapiro–Wilk test statistics W ranged from 0.95 to 0.98, all *P*-values > 0.05), justifying the use of parametric tests. Therefore, continuous variables are presented as means ± standard deviations (M ± SD), and group comparisons were conducted using independent samples t-tests. Categorical variables are presented as frequencies and percentages [n (%)] and were compared between groups using the *χ*² test. Pearson correlation analysis was used to examine the relationships between caregiver psychological resilience, family function, and uncontrolled asthma in children. The significance level was set at α = 0.05.

## 3 Results

### 3.1 General information

Demographic and disease-related characteristics, family and social factors between the controlled group (*n* = 60) and uncontrolled group (*n* = 52) were analyzed (Table 1). No significant differences were observed in age (*P* = 0.759), gender distribution (*P* = 0.797), BMI percentile (*P* = 0.168), allergy history (*P* = 0.525), disease duration (*P* = 0.108), regular follow-up (*P* = 0.496), being an only child (*P* = 0.765), and school attendance (*P* = 0.137). This similarity enhances the comparability of the groups for evaluating psychosocial factors.

**Table 1:** Comparison of children situation between the two groups.

Parameters	Controlled group (*n* = 60)	Uncontrolled group (*n* = 52)	t/*χ*^2^	*P*
Demographics				
Age (years)	9.03 ± 0.95	9.08 ± 0.92	0.308	0.759
Gender [*n* (%)]			0.066	0.797
Male	39 (65.00%)	35 (67.31%)		
Female	21 (35.00%)	17 (32.69%)		
BMI percentile (%)	75.42 ± 10.15	77.91 ± 8.67	1.388	0.168
Disease-related Characteristics				
Allergy history [*n* (%)]			0.405	0.525
Yes	48 (80.00%)	44 (84.62%)		
No	12 (20.00%)	8 (15.38%)		
Disease duration (months)	25.45 ± 8.36	28.21 ± 9.67	1.621	0.108
Regular follow-up [*n* (%)]			0.463	0.496
Yes	45 (75.00%)	36 (69.23%)		
No	15 (25.00%)	16 (30.77%)		
Family and social factors				
Only child [*n* (%)]			0.090	0.765
Yes	34 (56.67%)	28 (53.85%)		
No	26 (43.33%)	24 (46.15%)		
School attendance [*n* (%)]			2.213	0.137
Attending school normally	56 (93.33%)	44 (84.62%)		
Frequent absence due to asthma	4 (6.67%)	8 (15.38%)		

BMI, body mass index (expressed as age- and sex-specific percentile).

In comparing the family situations between the controlled group and uncontrolled group of children with asthma, our analysis revealed no significant differences in primary caregiver identity (*P* = 0.692), mother's age (*P* = 0.489), mother's education level (*P* = 0.636), father's age (*P* = 0.615), father's education level (*P* = 0.645), family structure (*P* = 0.482), monthly family income (*P* = 0.390), and family member smoking status (*P* = 0.821) (Table 2). This indicates that the groups were comparable in socioeconomic and environmental backgrounds, allowing for a clearer focus on caregiver and family function differences.

**Table 2:** Comparison of family situation between the two groups.

Parameters	Controlled group (*n* = 60)	Uncontrolled group (*n* = 52)	t/*χ*^2^	*P*
Primary caregiver identity [*n* (%)]			0.157	0.692
Mother	48 (80.00%)	40 (76.92%)		
Father	12 (20.00%)	12 (23.08%)		
Mother				
Age (years)	36.45 ± 4.12	37.02 ± 4.55	0.694	0.489
Education level [*n* (%)]			0.904	0.636
High school or below	18 (30.00%)	20 (38.46%)		
College or Bachelor's degree	35 (58.33%)	27 (51.92%)		
Master's degree or above	7 (11.67%)	5 (9.62%)		
Father				
Age (years)	38.67 ± 4.89	39.15 ± 5.21	0.504	0.615
Education level [*n* (%)]			0.876	0.645
High school or below	20 (33.33%)	21 (40.38%)		
College or Bachelor's degree	33 (55.00%)	27 (51.92%)		
Master's degree or above	7 (11.67%)	4 (7.69%)		
Family environment and resources				
Family structure [*n* (%)]			1.462	0.482
Nuclear family	39 (65.00%)	28 (53.85%)		
Stem family	19 (31.67%)	22 (42.31%)		
Single parent	2 (3.33%)	2 (3.85%)		
Monthly family income [*n* (%)]			1.883	0.390
<10,000 RMB	10 (16.67%)	14 (26.92%)		
10,000–20,000 RMB	35 (58.33%)	28 (53.85%)		
>20,000 RMB	15 (25.00%)	10 (19.23%)		
Family member smoking [*n* (%)]			0.051	0.821
Yes	22 (36.67%)	18 (34.62%)		
No	38 (63.33%)	34 (65.38%)		

### 3.2 Caregiver psychological resilience

**Figure 1:**
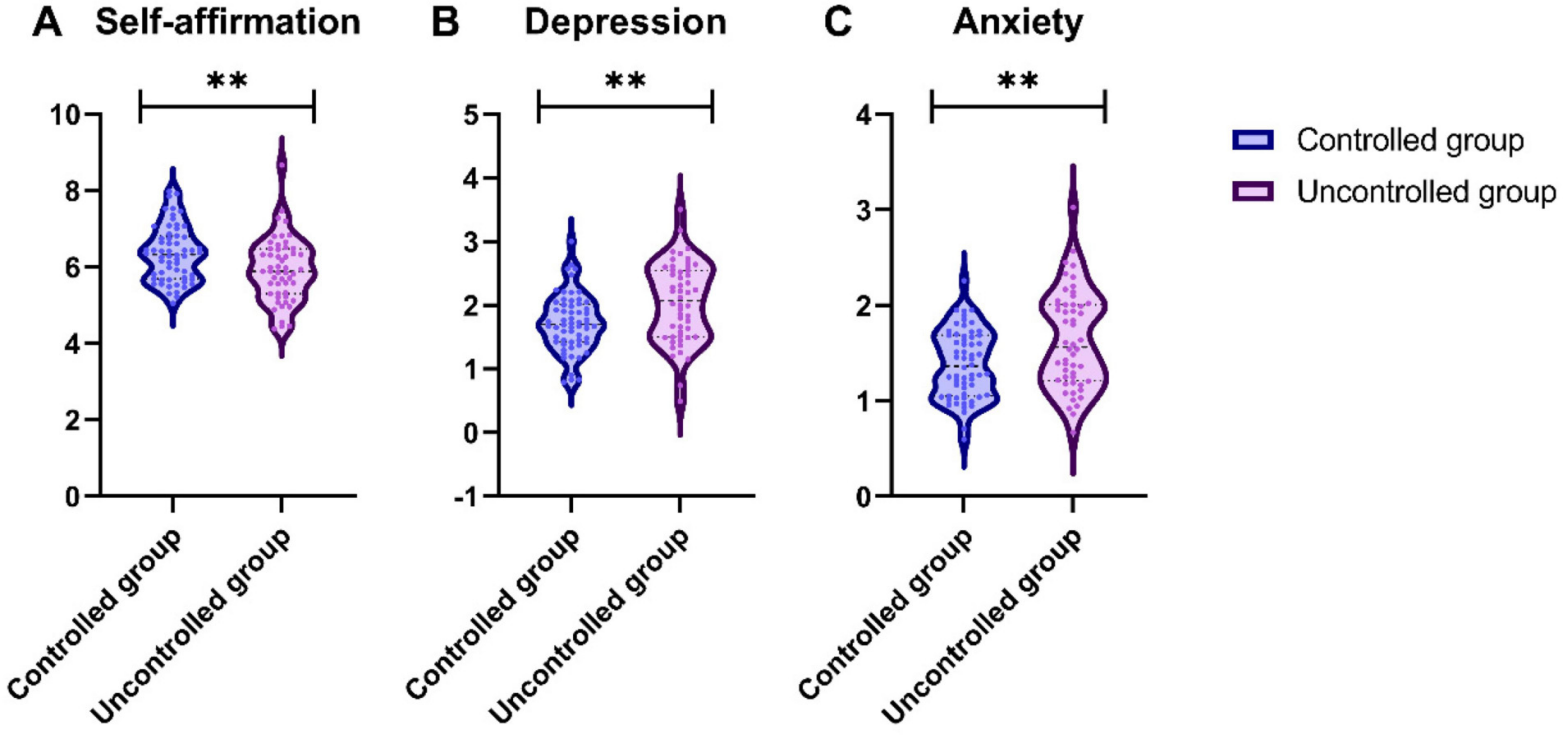
Comparison of general health questionnaire (GHQ) between the two groups (scores). **(A)** Self-affirmation; **(B)** Depression; **(C)** Anxiety; **: *P* < 0.01.

The comparison of GHQ scores between the controlled group and uncontrolled group revealed significant differences in several psychological parameters (Figure 1). Specifically, the controlled group exhibited a significantly higher score in self-affirmation (*t* = 2.910, *P* = 0.004). Conversely, the uncontrolled group reported significantly higher scores in depression (*t* = 3.093, *P* = 0.003) and anxiety (*t* = 3.007, *P* = 0.003).

In the comparison of CHIP scores between the controlled group and uncontrolled group, significant differences were observed across several domains (Table 3). Specifically, the controlled group scored significantly higher in family coping strategies (*t* = 2.821, *P* = 0.006), support resources (*t* = 2.250, *P* = 0.026), and medical management skills (*t* = 2.533, *P* = 0.013).

**Table 3:** Comparison of coping health inventory for parents (CHIP) between the two groups (scores).

Parameters	Controlled group (*n* = 60)	Uncontrolled group (*n* = 52)	*t*	*P*
Family	45.25 ± 4.12	42.81 ± 5.03	2.821	0.006
Support	49.83 ± 3.67	48.15 ± 4.22	2.250	0.026
Medical	32.45 ± 3.89	30.44 ± 4.51	2.533	0.013

### 3.3 Family function

The comparison of FAD scores between the controlled group and uncontrolled group revealed significant differences across multiple domains (Table 4). Specifically, the uncontrolled group scored significantly higher in problem-solving (*t* = 2.386, *P* = 0.019), communication (*t* = 3.079, *P* = 0.003), roles (*t* = 2.549, *P* = 0.012), affective responsiveness (*t* = 3.009, *P* = 0.003), affective involvement (*t* = 3.236, *P* = 0.002), behavioral control (*t* = 2.154, *P* = 0.034), and general functioning (*t* = 2.229, *P* = 0.028).

**Table 4:** Comparison of family assessment device (FAD) between the two groups (scores).

Parameters	Controlled group (*n* = 60)	Uncontrolled group (*n* = 52)	*t*	*P*
Problem-solving	2.29 ± 0.25	2.41 ± 0.27	2.386	0.019
Communication	2.06 ± 0.22	2.19 ± 0.23	3.079	0.003
Roles	2.45 ± 0.18	2.53 ± 0.18	2.549	0.012
Affective responsiveness	2.58 ± 0.24	2.71 ± 0.21	3.009	0.003
Affective involvement	2.47 ± 0.31	2.61 ± 0.14	3.236	0.002
Behavioral control	2.57 ± 0.22	2.65 ± 0.14	2.154	0.034
General functioning	2.49 ± 0.17	2.57 ± 0.19	2.229	0.028

**Figure 2:**
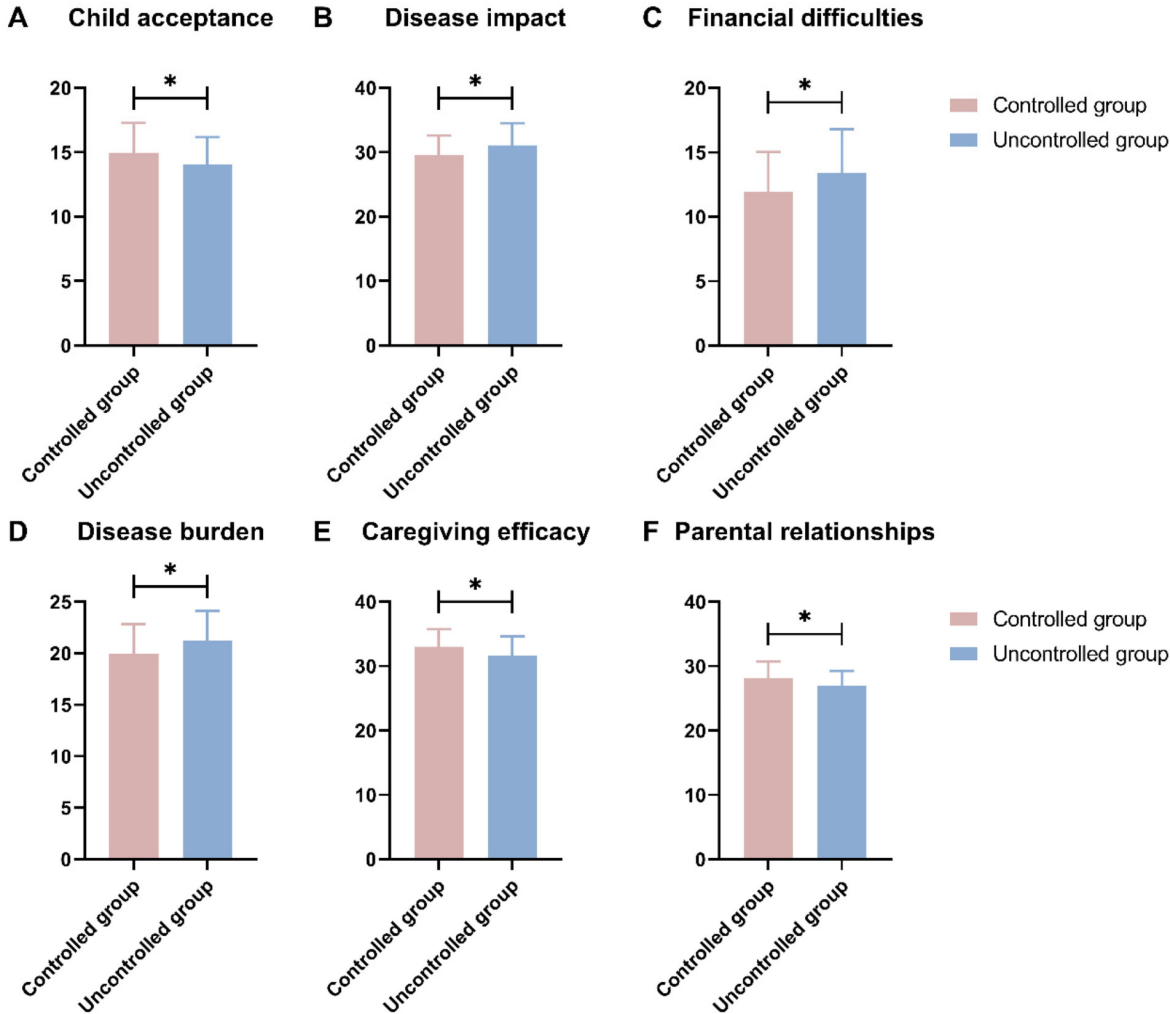
Comparison of family management measure (FaMM) between the two groups (scores). **(A)** Child acceptance; **(B)** Disease impact; **(C)** Financial difficulties; **(D)** Disease burden; **(E)** Caregiving efficacy; **(F)** Parental relationships; *: *P* < 0.05.

In the comparison of FaMM scores between the controlled group and uncontrolled group, significant differences were observed in several key areas (Figure 2). Specifically, the controlled group scored significantly higher in child acceptance (*t* = 2.194, *P* = 0.030) and caregiving efficacy (*t* = 2.445, *P* = 0.016), while also scoring lower in disease impact (*t* = 2.530, *P* = 0.013), financial difficulties (*t* = 2.364, *P* = 0.020), disease burden (*t* = 2.370, *P* = 0.020), and parental relationships (*t* = 2.350, *P* = 0.021).

### 3.4 Correlation analysis

The correlation analysis between caregiver psychological resilience and uncontrolled asthma in children revealed several significant relationships (Table 5). Specifically, self-affirmation scores from the GHQ were negatively correlated with uncontrolled asthma (*r* = −0.249, *P* = 0.008). Conversely, depression (*r* = 0.277, *P* = 0.003) and anxiety (*r* = 0.252, *P* = 0.007) scores from the GHQ were positively correlated with uncontrolled asthma. Additionally, CHIP scores for family coping strategies (*r* = −0.258, *P* = 0.006), support resources (*r* = −0.212, *P* = 0.025), and medical management skills (*r* = −0.241, *P* = 0.010) were negatively correlated with uncontrolled asthma.

**Table 5:** Correlation analysis between caregiver psychological resilience and uncontrolled asthma in children.

Parameters	r	*P*
Self-affirmation (GHQ)	−0.249	0.008
Depression (GHQ)	0.277	0.003
Anxiety (GHQ)	0.252	0.007
Family (CHIP)	−0.258	0.006
Support (CHIP)	−0.212	0.025
Medical (CHIP)	−0.241	0.010

GHQ, general health questionnaire; CHIP, coping health inventory for parents.

The correlation analysis between family function and uncontrolled asthma in children reveals several significant relationships (Table 6). In terms of the FAD, all assessed domains showed positive correlations with uncontrolled asthma: problem-solving (*r* = 0.232, *P* = 0.014), communication (*r* = 0.245, *P* = 0.009), roles (*r* = 0.219, *P* = 0.020), affective responsiveness (*r* = 0.277, *P* = 0.003), affective involvement (*r* = 0.248, *P* = 0.008), behavioral control (*r* = 0.216, *P* = 0.022), and general functioning (*r* = 0.194, *P* = 0.041). Conversely, for the FaMM, child acceptance (*r* = −0.207, *P* = 0.029) and caregiving efficacy (*r* = −0.200, *P* = 0.034) were negatively correlated with uncontrolled asthma. On the other hand, disease impact (*r* = 0.223, *P* = 0.018), financial difficulties (*r* = 0.191, *P* = 0.044), disease burden (*r* = 0.209, *P* = 0.027), and parental relationships (*r* = −0.207, *P* = 0.029) also showed significant correlations.

**Table 6:** Correlation analysis between family function and uncontrolled asthma in children.

Parameters	r	*P*
Problem-solving (FAD)	0.232	0.014
Communication (FAD)	0.245	0.009
Roles (FAD)	0.219	0.020
Affective responsiveness (FAD)	0.277	0.003
Affective involvement (FAD)	0.248	0.008
Behavioral control (FAD)	0.216	0.022
General functioning (FAD)	0.194	0.041
Child acceptance (FaMM)	−0.207	0.029
Disease impact (FaMM)	0.223	0.018
Financial difficulties (FaMM)	0.191	0.044
Disease burden (FaMM)	0.209	0.027
Caregiving efficacy (FaMM)	−0.200	0.034
Parental relationships (FaMM)	−0.207	0.029

FAD, family assessment device; FaMM, family management measure.

## 4 Discussion

This study investigated the relationships between disease control in children with asthma and the psychological resilience of their caregivers as well as overall family functioning. The findings reveal marked differences between the controlled and uncontrolled asthma groups across multiple dimensions of caregiver psychology and family dynamics, with no observed differences in general demographic or family characteristics. This suggests that psychosocial factors show a stronger association with asthma control outcomes than socioeconomic or basic demographic variables in this cohort. However, given the study design, this association may reflect a bidirectional relationship, where asthma status and psychosocial factors mutually influence each other.

### 4.1 Caregiver psychological resilience and asthma control

Our analysis indicates that caregivers of children with well-controlled asthma reported higher levels of self-affirmation and lower levels of depression and anxiety. Conversely, caregivers in the uncontrolled group experienced more pronounced negative emotions. This aligns with a recent qualitative study which identified that caregivers of children with severe asthma often bear a substantial emotional burden, including feelings of fear, nervousness, and worry (20). The constant vigilance required for symptom monitoring, environmental trigger management, and medication adherence can be a chronic source of stress, depleting caregivers' psychological resources (21).

The mechanism linking caregiver mental state to child health outcomes may involve several pathways. Firstly, a caregiver experiencing depression or anxiety may have reduced capacity to consistently execute complex management plans, potentially leading to missed medications or delayed responses to symptoms (22). Secondly, caregiver stress can create a tense home environment, which may psychologically affect the child and potentially exacerbate their asthma symptoms through psychoneuroimmunological pathways (23). This is supported by prior research indicating that higher levels of family chaos are significantly associated with poorer asthma control in children (24). Furthermore, our correlation analysis supports a negative relationship between caregiver self-affirmation and uncontrolled asthma, suggesting that a positive, confident mindset in caregivers is a protective factor for disease management. This is corroborated by other research showing that interventions providing support, such as from community health workers, can alleviate caregiver stress and simultaneously improve pediatric asthma control (23, 25).

### 4.2 Parental coping strategies as a buffer

In the domain of coping strategies, our study found that caregivers in the controlled group utilized more effective coping mechanisms related to family integration, social support, and medical management. The CHIP measures practical efforts to maintain family cohesion, seek emotional and instrumental support, and actively engage with healthcare systems. The lower scores in the uncontrolled group suggest a depletion of these vital coping resources (23, 26).

This finding resonates with the concept that effective coping mitigates burden. The identified burdens in severe pediatric asthma—such as disrupted sleep, lack of support at school, and constant time demands—require robust coping strategies to overcome (27, 28). When caregivers are unable to employ these strategies, the caregiving process is hindered. The positive correlation we observed between effective coping and better asthma control implies that these strategies are not merely beneficial for the caregiver's well-being but are directly consequential for the child's health (29). Interventions grounded in self-efficacy theory posit that supporting caregivers' confidence and competence in management tasks can improve adherence and outcomes. Our results affirm that comprehensive coping, encompassing family, social, and medical domains, is a critical component of this process (13).

### 4.3 The central role of family function and disease management

Perhaps the most pronounced differences between the groups were observed in family function and disease management. Using the FAD, we found that families of children with uncontrolled asthma exhibited greater dysfunction across all measured domains, including problem-solving, communication, affective involvement, and behavioral control. Poor problem-solving can lead to ineffective responses to asthma exacerbations, while impaired communication hinders the clear relay of symptoms and needs between the child and caregivers. Dysfunctional affective involvement and responsiveness may result in either insufficient emotional support or excessive anxiety around the child's condition, neither of which is conducive to optimal management (12, 30).

This picture of family dysfunction is further detailed by the FaMM. Families in the uncontrolled group perceived a greater negative impact of the disease on the family, a higher financial and daily burden, and lower efficacy in their caregiving abilities. They also reported less mutual support in their parental relationships. Critically, these families demonstrated lower levels of child acceptance, which may reflect difficulties in integrating the reality of a chronic illness into family life. The correlation between these factors and poorer asthma control underscores that asthma management is a family systems issue, not just an individual one (31). A Chinese study similarly highlighted that family management practices, such as the parents' ability to manage respiratory infections and adhere to medications, are closely tied to asthma control levels (32, 33). Our findings confirm that the overall health of the family system, as reflected in the FAD and FaMM, is a fundamental determinant of these management capabilities.

### 4.4 The interplay of factors and clinical implications

The results collectively paint a coherent picture of a bidirectional relationship: the burden of pediatric asthma may challenge caregiver mental health and strain family systems (34), while conversely, diminished caregiver resilience and compromised family function could impede the consistent, high-quality management required for good asthma control (35). This creates a potential negative feedback loop, where poorer asthma control and psychosocial difficulties exacerbate each other over time. Our correlation analyses strengthen this interpretation, showing that poorer psychological resilience in caregivers and worse family function are consistently associated with uncontrolled asthma (9).

A promising approach to breaking this cycle, as suggested by emerging research, is to focus on enhancing family resilience. This involves strengthening the family's ability to adapt to the challenges of chronic illness collectively (36). Furthermore, a study has concluded that psychosocial interventions can have beneficial effects on both asthma symptoms and parental stress in families of school-age children (20). Our findings provide specific targets for such interventions: supporting caregiver mental health (particularly targeting depression, anxiety, and self-affirmation), teaching effective coping strategies (especially around family integration and medical management), and improving key family functional domains like communication, problem-solving, and affective involvement (35).

### 4.5 Limitations and future directions

This study has several limitations. Its cross-sectional design does not allow us to establish causality. While we posit that caregiver resilience and family function influence asthma control, it is equally plausible that poor asthma control in the child leads to worsened caregiver mental health and family strain. Thus, the observed associations should be interpreted as bidirectional correlations, with no definitive causal direction inferred from this study. The sample was recruited from a single hospital, which may limit the generalizability of the findings to other populations or healthcare settings. Furthermore, the reliance on self-reported questionnaires is subject to biases, such as social desirability bias. Future research should employ longitudinal designs to trace the temporal relationships between these variables and clarify causal pathways. Intervention studies are needed to test whether programs aimed at boosting caregiver psychological resilience and improving family function directly lead to better pediatric asthma control outcomes. These interventions could be based on approaches like acceptance and commitment therapy (ACT), which has been shown to improve parental psychological flexibility and family functioning in challenging contexts (29). Finally, exploring these dynamics in more diverse populations and including objective measures of asthma control, such as lung function tests, would strengthen the validity and impact of the findings.

## 5 Conclusion

In summary, this study demonstrates a significant bidirectional association between poorer asthma control in children, lower psychological resilience among caregivers, and more dysfunctional family dynamics. While the direction of causality remains unclear due to the cross-sectional design, these findings highlight the interdependence of these factors in asthma management. Caregivers of children with uncontrolled asthma exhibited more negative psychological states and less effective coping strategies, while their families showed greater dysfunction across multiple domains and less optimal disease management approaches. These findings underscore that pediatric asthma control is not solely a biomedical issue but is deeply intertwined with the psychosocial health of the caregiver and the functional capacity of the family unit. A comprehensive approach to asthma management should therefore integrate strategies to support caregiver mental health and strengthen family functioning, potentially leading to improved health outcomes for children with this chronic condition.

## Data Availability

The raw data supporting the conclusions of this article will be made available by the authors, without undue reservation.
